# "It's hard to tell": The challenges of scoring patients on standardised outcome measures by multidisciplinary teams: a case study of neurorehabilitation

**DOI:** 10.1186/1472-6963-8-217

**Published:** 2008-10-22

**Authors:** J Greenhalgh, AF Long, R Flynn, S Tyson

**Affiliations:** 1 School of Healthcare, University of Leeds, Baines Wing, Woodhouse Lane, Leeds, LS2 9JT, UK; 2 Institute of Social, Cultural and Policy Research, University of Salford, Humphrey Booth House, Salford, M5 4WT, UK; 3 Centre for Rehabilitation and Human Performance Research, University of Salford, Mary Seacole Building, Salford, UK

## Abstract

**Background:**

Interest is increasing in the application of standardised outcome measures in clinical practice. Measures designed for use in research may not be sufficiently precise to be used in monitoring individual patients. However, little is known about how clinicians and in particular, multidisciplinary teams, score patients using these measures. This paper explores the challenges faced by multidisciplinary teams in allocating scores on standardised outcome measures in clinical practice.

**Methods:**

Qualitative case study of an inpatient neurorehabilitation team who routinely collected standardised outcome measures on their patients. Data were collected using non participant observation, fieldnotes and tape recordings of 16 multidisciplinary team meetings during which the measures were recited and scored. Eleven clinicians from a range of different professions were also interviewed. Data were analysed used grounded theory techniques.

**Results:**

We identified a number of instances where scoring the patient was 'problematic'. In 'problematic' scoring, the scores were uncertain and subject to revision and adjustment. They sometimes required negotiation to agree on a shared understanding of concepts to be measured and the guidelines for scoring. Several factors gave rise to this problematic scoring. Team members' knowledge about patients' problems changed over time so that initial scores had to be revised or dismissed, creating an impression of deterioration when none had occurred. Patients had complex problems which could not easily be distinguished from each other and patients themselves varied in their ability to perform tasks over time and across different settings. Team members from different professions worked with patients in different ways and had different perspectives on patients' problems. This was particularly an issue in the scoring of concepts such as anxiety, depression, orientation, social integration and cognitive problems.

**Conclusion:**

From a psychometric perspective these problems would raise questions about the validity, reliability and responsiveness of the scores. However, from a clinical perspective, such characteristics are an inherent part of clinical judgement and reasoning. It is important to highlight the challenges faced by multidisciplinary teams in scoring patients on standardised outcome measures but it would be unwarranted to conclude that such challenges imply that these measures should not be used in clinical practice for decision making about individual patients. However, our findings do raise some concerns about the use of such measures for performance management.

## Background

### The rise of outcome measurement in clinical practice

Since the 1970's, healthcare policy across Europe and the US has involved a move towards evidence based practice [1,2]. A key component of these policy shifts has been a focus away from the measurement of the structure and process of care, towards an assessment of outcome as indicator of the effectiveness or quality of care [3,4]. Alongside this, and in part driven by this focus on outcome, there has been an exponential growth in the development of a large range of standardised outcome measures [5,6]. These measures are designed to be completed by either practitioners or by patients in order to measure constructs such as impairment, functioning, participation, health status and health related quality of life. These measures are most commonly used in clinical trials of the effectiveness of care and treatment [7,8]. Recently, their use in clinical practice has also been advocated [9-11]. Such data are argued to have a role in facilitating decision making about individual patients [12], enabling clinicians to identify whether their treatments are working [13] and, at an aggregated level, to facilitate comparisons of the effectiveness between different treatments, services and institutions [14-16].

The literature on standardised outcome measures is dominated by quantitative statistical assessments of their psychometric properties, namely, validity, reliability and responsiveness to change [17]. Measures must meet these criteria in order to reassure both practitioners and the research community that standardised outcome measures yield accurate, trustworthy information. Such evidence also forms the basis on which instruments are deemed appropriate for use in research [18] and clinical practice [19]. The evidence to support the psychometric properties of standardised outcome measures is often obtained from research studies, rather than the 'messiness' of real life clinical practice. Furthermore, most instruments were designed for use in research settings, rather than clinical practice. As such, some have argued that research based measures lack the precision required for monitoring individual patients in clinical practice [7]. Others question whether clinicians, who have a stake in the outcome of the intervention, are in a position to assess such outcomes objectively [20].

This raises questions about how clinicians, particularly those working in multidisciplinary teams, apply clinician rated measures within clinical practice and the extent to which real life clinical practice reflects how the measures are assessed within psychometric studies or used in research contexts. Such teams are the norm in the care of patients with chronic diseases and the use of standardised outcome measures has been proposed as one means of facilitating communication within these teams and of improving outcomes for patients [21,22]. In this paper, we consider two challenges of using outcome measures in clinical practice by multidisciplinary teams. The first relates to the reality of using standardised outcome measures in the clinical context and the second concerns the process of achieving a shared understanding of scoring guidelines amongst clinicians from different professional backgrounds when working with patients with severe and multiple complex problems.

### Psychometrics: research vs clinical practice

Standardised outcome measures themselves usually consist of a number of different items assessing different aspects of the patient's functioning. This assumes that a patient's problems can be divided up into different domains and each domain rated separately and independently of another. However, in clinical practice, patients often have multiple problems which may be difficult to separate out and may influence each other [23].

In studies evaluating inter-rater reliability, clinicians are trained in the use of the scoring guidelines and time is set aside for them to assess the patient in a specific setting and then to rate them on the measure. In research studies, ratings are often undertaken by a single independent observer who is not directly involved in providing the patient's care. Thus, the degree of familiarity that raters have with patients is similar both between raters and across patients and all raters rate the patient in the same setting. This may not be the case in clinical practice, where the social organisation of work across different professions leads to these practitioners working in different ways and in different settings with patients, which, in turn, provides them with a different knowledge base about that patient [24,25]. Thus it is possible that different members of staff may have equally valid but different judgements about the patient's functioning and the knowledge to score the measure may be distributed across different clinicians. In research settings, raters with different opinions are treated as sources of error. However, in clinical practice, differences of opinion must be resolved and distributed knowledge brought together, but we know little about how this is achieved.

In evaluations of test re-test reliability, raters assess the patient on two occasions, separated by an interval long enough such that raters are not influenced by their original ratings but short enough to ensure that the patient's functioning has not changed. In studies evaluating treatment effectiveness, independent observers rarely meet patients in between ratings and do not work directly with them. Thus, the rater's familiarity with the patient does not change across the two occasions. However, in clinical practice, as clinicians develop a working relationship with patients over time, their understanding of the patient's problems may change. Judgements made in the early stages of this relationship may thus be subject to revision and adjustment. From a psychometric standpoint, such adjustments would again be considered as sources of error and jeopardise an instrument's responsiveness to change. In the context of clinical practice, such adjustments are a necessary and integral part of clinical judgement [26].

### Standardisation and subjective interpretation

One of the much cited advantages of outcome measures is their standardised nature. Since all respondents are asked the same questions or rated using the same criteria and their answers calibrated using the same metric, this, it is argued, ensures that the resulting scores can be compared across groups or individuals or within groups or individuals over time. However, despite care in the development of measures, there is much debate about whether standardised questions and response options necessarily lead to raters or respondents interpreting them in the same way [23,27]. A small number of qualitative studies have revealed problems with the subjective interpretation of standardised health measures that would call into question their validity and reliability, despite quantitative evidence to support these properties [23,27-31].

Clear scoring guidelines and training of raters aim to ensure standardisation in their interpretation and application [32]. However, such guidelines are not always sufficient to resolve disputes over score allocation amongst a team of clinicians [33]. Practitioners from different professions may work on the basis of different models of health and illness in making judgements about patients [34,35], which are the very concepts addressed by standardised outcome measures. Thus, practitioners may differ in their interpretation of the concepts addressed within the standardised measure and the guidelines to score them. This raises questions about how multidisciplinary teams accomplish a shared meaning in the concepts addressed by standardised outcome measures when applying standardised outcome measures in practice.

In this paper, we explore how a multidisciplinary team of clinicians achieved the task of allocating scores to patients using a series of standardised outcome measures. We aim to examine the challenges they faced in accomplishing this task.

## Methods

### Setting

This paper is based on field work from an ESRC funded study that used neurorehabilitation as a case study to explore how, and to what extent, multidisciplinary teams used standardised outcome measures in clinical decision making. To preserve anonymity, we have changed the names of the unit, patients, staff and locations mentioned throughout the paper. Around 85% of neurorehabilitation teams in the UK collect at least one standardised outcome measure as part of routine patient care [36]. These teams include clinicians from a range of professional backgrounds, including medical, nursing, physiotherapy, occupational therapy, social work and neuropsychology who work using different models of health and illness. As such, they provide a valuable context in which to explore how different professional groups make sense of and apply standardised outcome measures to their patients.

We purposively selected one in-patient neurorehabilitation team based at the Churchtown Centre who routinely collected a number of standardised outcome measures to take part in this study. The 19 bed unit was at a satellite site of a large NHS teaching hospital and provided in-patient neurorehabilitation for patients aged between 16 and 65. Access to the Churchtown Centre was initially discussed with the lead consultants and then approved by the whole team. We obtained University and NHS ethical approval and all members of staff gave written informed consent to take part.

### Data collection

Data were collected by one member of the research team (JG) using non-participant observation and semi-structured interviews [37]. Our data collection was focused on the regular MDT meetings in which the standardised outcome measures were recited and scored by the team. Two MDT meetings lasting one-and-a-half hours were held each week and each was led by one of two Consultants. A total of 16 MDT meetings were attended and observed, and 14 of these meetings were fully audio-taped and transcribed. The recordings for two meetings were not transcribed because they were inaudible; one meeting was held in a different room with team members sat some distance from each other and the microphone was not able to detect all the voices. In the other instance the batteries failed in the microphone.

During the fieldwork, 39 patients were considered by the team. Towards the end of the field work, we interviewed at least one clinician from each of the professional groups in the team, including the two consultants (n = 11). These interviews were audio-taped and transcribed. Field notes were taken throughout data collection and distributed amongst the research team for ongoing analysis. A memo was kept to record analytical insights and any possible changes to usual team behaviour as a result of JG's presence within MDT meetings. These changes were explored by noting down instances when team members commented on JG's presence or the presence of the tape recorder at the meeting or spoke directly to JG to ask questions or explain or justify anything that had been said. Our analysis suggested that the team's explicit awareness of JG's presence during meetings was greatest in the first three meetings but this awareness did not appear to lead to any editing or curtailing of discussions.

### Analysis

Qualitative data analysis was iterative and ongoing throughout the study using the techniques of grounded theory [38,39] and was aided by QSR Nvivo v2.0. Emerging themes were discussed and refined through regular meetings amongst the research team.

Based on repeated readings of the field notes, memos and transcripts of MDT meetings we initially developed broad open codes to describe how the process of 'scoring' was organised amongst the team. Three important properties of 'scoring' that emerged from this analysis related to the certainty or uncertainty about the score, disagreements amongst the team about the score, and ways of resolving uncertainty and disagreements, either individually or through group discussion. Combining these themes through axial coding led to us to conceptualise the process of scoring as taking on two overall forms, which we termed 'unproblematic' and 'problematic'.

Alongside this we analysed the interview data to identify the challenges or difficulties clinicians experienced in the process of scoring and the factors that influenced these. We then integrated the themes that emerged from the MDT meetings and interviews using selective coding to explain why the different types of scoring occurred. This involved (1) looking at the scores themselves to identify which items were more likely to be questioned, give rise to uncertainty or require a group discussion to agree on a score and which were not and (2) exploring when the two forms of scoring occurred within the MDT meetings depending on the contextual factors identified within the interviews. We also explored what was being disputed when discussion was necessary in the process of allocating scores to patients. In doing so we show how the production of the scores was organised and highlighted some of the challenges the team faced in producing them.

## Results

### Context

In order to place our analyses in context, some background information is provided about the structure of the MDT meetings and the team's use of outcome measures. The MDT meetings were usually led by one of two consultants and were also attended by a registrar and/or senior house officer (SHO), who were present throughout the meeting. During the study two different SHOs were on rotation in the unit. At each meeting, six or seven patients were discussed and each was allocated a 15 minute timeslot. While there were almost always representatives from the nursing, occupational therapy and physiotherapy staff, the individuals in attendance changed for each patient, depending on which member of the therapy staff was taking the lead on that person's care, or which nurses were working in the bay in which the patient was staying. Other staff attended either when a patient with whom they were working with was to be discussed (the speech therapist and the clinical neuropsychologist) or when their workload allowed (family liaison health visitor). Three social workers were attached to the team and attended meetings when discharge arrangements were being finalised.

The analysis on which this paper is based focused on the measures that were recited and sometimes actually 'scored' within the MDT meetings themselves. This 'shared scoring' usually occurred at the beginning of the discussion for each patient. The standardised measures assessed a range of concepts across the WHO International Classification of functioning, disability and health (ICF) and included psychometrically assessed measures namely, the Barthel Index [32,40], the Northwick Park Dependency Score (NPDS) [41], the Leeds Handicap Scale [42] and the Waterlow Score [43]. They also included a set of 'homegrown' single item scores that had not been psychometrically assessed but nonetheless required team members to agree which category the patient fell into. These measured problems that both reflected the patient's progress in rehabilitation but were also indicators of potential barriers to progress such as memory, concentration, confusion, motivation, anxiety and depression. Table 1 provides a summary of the measures used. As the clarity or otherwise of the Barthel Index is discussed extensively within this paper, a more detailed description of three examples of items from the version used by team [44] is provided in Table 2.

**Table 1:** Summary of measures used at the Churchtown centre

Name	Content	Scoring
Barthel Index	Measure of activities of daily living. The Churchtown centre used the modified Barthel Index [44]. Contains ten items measuring: personal hygiene, bathing, feeding, toileting, stair climbing, dressing, bowel control, bladder control, ambulation, chair/bed transfers, plus an additional item for wheelchair users.	The amount of assistance required for the patient to perform each item is scored at one of five levels. Different descriptors, or 'scoring guidelines' are provided for each level of each item. The numerical score for each level varies for each item, for example, personal hygiene is scored at 0,1,3,4,5; Feeding is scored 0,2,5,8,10; ambulation is scored 0,3,8,12,15. The total scores range from 0 to 100. Lower scores indicate a greater need for assistance.

Northwick Park Dependency Score	Assesses the impact of patient dependency on nursing time. Consists of two sections. The Basic Care Needs has 16 items measuring mobility, bed transfers, toileting bladder, urinary incontinence, toileting bowels, faecal incontinence, washing and grooming, bathing/showering, dressing, eating, drinking, enteral feeding, skin pressure relief, safety awareness, communication and behaviour. The Special Nursing Needs section has 7 items covering tracheostomy, open wound requiring dressing, requires > 2 interventions at night, requires psychological support, in isolation, acute medical/surgical intervention, needs one to one 'specialing'.	Items on the basic care needs section are scored according to the amount of help required to perform the tasks which, for some items, includes and indication of the amount of nursing time needed to provide this help. Items are scored on either a 0–3, 0–4 or 0–5 range, depending on the different specifications of amount of helped needed for each items. Items scores are summed to give a range between 0 and 65. Special care needs are dichotomous variables scored at either 0 (not present) or 5(present) and are then summed to give a total score from 0 to 35. The two scores are then added together to give a composite NPDS ranging from 0 to 100 with higher scores indicating the need for more help.

The Leeds assessment scale of handicap	Assess four of the six 'survival' roles in the WHO definition of handicap (now participation): mobility, physical independence, orientation and social integration	Each item is scored nine point scale from 0 to 8, with 0 indicating the highest level of fulfilment of the role. Different descriptors, or 'scoring guidelines' are provided for each level of each item. For the 'orientation item', two descriptors each are provided for levels four and five.

Waterlow score	Measures risk of pressure scores. Consists of eight items representing different risk factors: appetite, continence, visual skin signs, mobility, build/weight, age, sex and special risks (eg (poor nutrition, sensory deprivation, high dosage of anti-inflammatory drugs, smoking, orthopaedic surgery)	For the first seven items, a single score is allocated on a different, graded scale for each item. These scores are then summed. Individual special risk factors are each allocated a score, which are also summed and added to the total for the first seven items.

'homegrown' scores	Single item assessments of language reception, language expression, functional communication, memory, concentration, confusion, drive/motivation, snacks and meals, anxiety, depression and behaviour.	Anxiety and depression: scored on a three point scale: 1: not present; 2: present but not affecting progress in rehabilitation; 3: present and affecting progress. Behaviour: six descriptors: normal, disinhibited, aggressive, disruptive, withdrawn, apathetic. Language: three items each scored on a five point scale with each item having different descriptors; Others: scored on a 5 point scale indicating the degree of problem in each area ranging from normal (5); slight but not affecting progress in rehabilitation (4); slight but affecting progress in rehabilitation (3); moderate (2) and severe (1).

**Table 2:** Details of scoring guidelines for three items from the Modified Barthel Index [44]

**Score**	**Guideline**
**Transfers **(usually scored by the physiotherapists)	

0	Unable to participate in a transfer. Two attendants are required with/without mechanical device.

3	Able to participate but maximum assistance of one other person is required in all aspects of the transfer.

8	The transfer requires the assistance of one other person in any aspect of transfer

12	The presence of another person is required, either as a confidence measure or to provide supervision for safety

15	The person can safely approach the bed/chair in a wheelchair, lock the brakes, left the footrests, move safely to bed, lie down, come to a sitting position on the side of the bed, change the position of the wheelchair, transfer back to it safely. The patient must be independent in all phases of this activity. Patient can come to a standing position if walking is the mode of locomotion. If walking, patient approaches, sits down, gets to a standing position from a regular chair, transfers from bed to chair, performs task safely.

**Feeding **(usually scored by the nurses)	

0	Dependent in all aspects and needs to be fed.

2	Can manipulate an eating device, usually a spoon, but someone must provide active assistance during the meal.

5	Able to feed self with supervision. Assistance is required with associated tasks, such as putting milk/sugar into tea, salt, pepper, spreading butter, turning a plate or other 'set up' activities.

8	Independence in feeding with prepared tray, except may need meat cutting, milk opening, jar lid opening etc. Presence of another person is not required.

10	The patient can feed self from a tray or table when someone puts the food within reach. The patient must put on an assistive device if needed, cut the food and if desired, use salt and pepper, spread butter etc.

**Dressing **(usually scored by the occupational therapist)	

0	The patient is dependent in all aspect of dressing and is unable to participate in the activity.

2	The patient is able to participate to some degree but is dependent in all aspects of dressing.

5	Assistance is needed in putting on and/or removing any clothing

8	Only minimal assistance is required with fastening clothing, such as buttons, zips, bra, shoes etc.

10	The patient should be able to put on/remove/fasten clothing, tie shoelaces, or put on/fasten/remove corset, braces, as prescribed.

It is important to note here that, although such measures are often termed 'outcome measures', team members rarely used them as such within MDT meetings. That is, they did not, at least explicitly, refer to the measures as providing evidence that rehabilitation was 'working' or used the measures judge the effectiveness of rehabilitation within the MDT meetings themselves. Instead, the team used to measures as 'tools' to predict likely progress at the outset of rehabilitation, monitor progress during rehabilitation and highlight the potential need for support at discharge. Within Kirshner and Guyatt's [45] framework of health indices, the measures were used by the team as both 'predictive' and 'evaluative' indexes. For such purposes, it is important that the measures have both adequate inter-observer reliability and test-retest reliability but are also able to detect real change when it occurs. In addition to its use in the MDT meetings, data from the NPDS was also being collected at the time of the fieldwork to provide the local Primary Care Trust (PCT) with evidence of the effectiveness of the unit and to make a case that the unit was in need of more occupational therapists. As such, the NPDS had a dual 'internal' and 'external' function for the MDT.

### Organisation of the scoring

Other writers have observed how the technical division of labour and professional segmentation of expertise differentially distributes knowledge about the patient within multidisciplinary teams [24,25]. Indeed, a challenge for multidisciplinary teams is to develop a 'shared picture' of the patient on which to establish common treatment goals [22]. Allocating scores on the standardised outcome measures used in the Churchtown Centre required knowledge about many different aspects of the patient's abilities and problems, which was determined by and reflected in the different types of work that team members did with patients by virtue of their professional background. This, in turn meant that the knowledge about the patient necessary for producing the scores was scattered amongst the team.

To address this, different items within the measures 'belonged' to a particular member of the team, based on their professional expertise, and she/he was responsible for producing that score for, and reporting it within the MDT. This division of labour can been seen below when the team are scoring Mr Spencer, a 64 year old man with a subarachnoid haemorrhage, two weeks after his admission to the unit.

Consultant 1 So, ok well let's do the Barthel.

(Long pause)

Physio Transfers eight. Ambulation nought. Wheelchair nought. Stairs nought.

SHO Feeding?

Nurse Umm feeding's ten.

[Consultant asks a medical student to take on the role of the SHO and read out the scores]

Medical student Dressing?

OT Five.

Nurse Yeah.

Medical student Personal hygiene?

OT Three.

Medical student Three?

OT Mmm.

Medical student Bathing?

OT Ahh one.

Medical student Using toilet?

(pause)

Nurse Uh two.

Medical student Urinary continent? Faecal continent?

Nurse Umm not a problem during the day and night.

SHO So that's ten

Nurse Ten sorry, yeah it's ten.

Medical student Ok. Language reception?

SALT Five.

Medical student Speech and language expression?

SALT Four.

Medical student Confusion? Oh I've missed one, function?

SALT Four.

Medical student For the functional or...?

SALT Function, yeah its five, four, four.

Here it is possible to see that the physiotherapist was seemingly responsible for the first four items of the Barthel Index (transfers, ambulation, wheelchair and stairs) while the nurse is responsible for the feeding, toileting and continence scores and the OT was responsible for the dressing, bathing and personal hygiene scores.

In their interviews team members explained that dividing the scores along professional boundaries was 'traditional' and was the quickest way to gain information about the patient.

"Erm, it's kind of traditionally done that way on (the) ward for some reason and, yes, you're more informed as a physio about whether (inaudible) independent scores in function and nursing staff are more informed about the continence side. So I guess it's that way, but erm if I haven't seen somebody with personal care then (inaudible) nursing staff to have their say 'cause they see that (inaudible)" Occupational therapist

"Erm, I wouldn't say that I would regard that [division of scoring] as a rule. It's more that it's traditional and it's the way we get the information quickest in the team meetings er. But, for example, we don't have demarcation disputes if it happens that either an OT or a nurse comments on dressing ability or something like that" Consultant

These quotes also reveal that this knowledge was not simply based on professional expertise but also on knowledge gained through the extent of team member's day to day work with the patient. Some scores, such as those in the Barthel Index, more clearly reflected the division of labour along professional boundaries, whilst others, such as those measuring handicap and 'homegrown' scores measuring concentration, memory, motivation and behaviour etc were an integral part of the work of all team members. These latter scores seemed to rely more heavily on team member's 'working knowledge' of the patient.

"Some of the mood ones or that kind of thing you can sort of comment on.... some of the other handicap ones it depends on who's in and how well they know them...We're not that sort of rigid and strict it depends on how well you know someone as to whether you can erm make a comment on you know like another area. Certainly to do with mood and behaviour and you know (inaudible) cognitive things you can comment on memory and motivation, concentration those kinds of things" (Physiotherapist)

Thus, the organisation of the production of the scores was determined by the division of labour amongst the team, both in terms of professional expertise and their working knowledge of particular patients. This is very different from how such scoring is organised in research settings, in which a single, independent observer is usually responsible for scoring all the items within a measure.

### Types of scoring: 'unproblematic' and 'problematic'

In many instances, the division of labour in the production of the scores enabled the differentially distributed knowledge about the patient to be brought together in straightforward manner. However, there were some instances in which it did not. We observed that the process of scoring the standardised outcome measures took two forms, which we have termed 'unproblematic' and 'problematic'. During 'unproblematic' scoring, the scores were simply reported by team members and it was tacitly assumed that there was no need for a discussion amongst team members to agree on a score. Extract 1 (above) provides an example of such unproblematic scoring. Here, the shared meaning of the scoring guidelines was a taken for granted aspect of the meeting [46]. 'Problematic' scoring was characterised by uncertainty and sometimes disagreement about the score, which called for discussion amongst the team members to reach a consensus.

**Figure 1:**
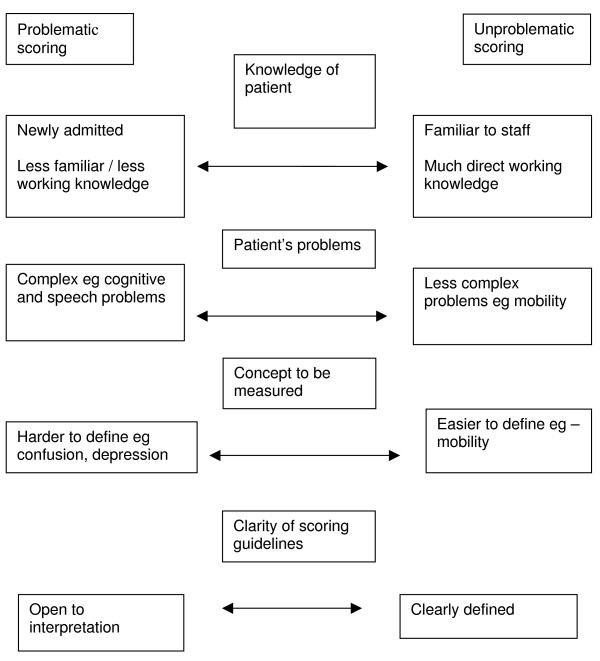
Factors affecting 'unproblematic' and 'problematic' scoring.

Our analyses identified a number of factors which gave rise to these two forms of scoring. These related to firstly, the team's and the practitioner's familiarity with the patient and the complexity of their problems and secondly, to the degree of complexity of the concepts to be measured and clarity of the scoring guidelines behind the scores themselves. In the next sections of the paper, we characterise these factors in more detail and show how they gave rise to the two different forms of scoring. Figure 1 represents our conceptualisation of these factors.

### Knowledge of the patient changing over time

'Problematic' scoring occurred most often when the patient was newly admitted to the unit. In these instances, the relevant therapy staff may have not had a chance to assess the patient and scoring the patient became more difficult and uncertain. Therapy staff often relied on the nurses to inform their scores because they saw the patient on a day to day basis, as the speech therapist explains below:

"I think it's more when people kind of first come and I've not had a chance to assess them and people will say 'well I don't think he's understanding things', and that I don't I can't really judge that so that's when I will say to the nurses is it affecting you know how he is, is it affecting how he's coping on the ward" (Speech therapist)

Some team members felt that scoring the patient in the first few weeks was sometimes based on 'guesswork' as the team did not yet have sufficient knowledge of the patient. The registrar acknowledged that

"the first, you know, one or two, are so inaccurate that you know, half of it's just guess work I suppose, you know, people putting their own scores, it's only later on that we really have a genuine feel of what they're doing with respect to the scores".

As the team became more familiar with the patient, the OT explained that "more information comes to light" so the team were "more informed" about the patient's problems and later scores then better reflected the true extent of patient's abilities and problems. However, a consequence of the initial 'guess work' was that, when comparing scores over time to judge the patient's progress, the patients' scores sometimes decreased when no real deterioration in the patient had taken place. This was apparent on the second occasion the team scored Mr Smith, a 63 year old man who had suffered a subarachnoid haemorrhage and traumatic brain injury resulting in significant cognitive problems. On the first occasion, the team had scored Mr. Smith a 'two' for memory, indicating that he had moderate problems.

Registrar Mmm. Memory

Consultant Does he recognise any staff?

Nurse I think he smiles, smiles (inaudible), I have to say good morning, he doesn't come over and

OT (inaudible)

Registrar Two is it moderate,

Nurse I'd say severe

OT Mmmm

Registrar It doesn't look good for scoring, he's down from two to one (laughing). Drive, initiation, motivation, scored a three last time, slight affecting progress

Nurse I wouldn't say its slight (pause). He doesn't initiate anything

Physio He hasn't got the cognition to have drive you know and self concept and (inaudible) so

Consultant He doesn't initiate anything does he

Physio No.

Consultant I mean apart from shuffling himself down in his chair

Registrar Erm I think we may have been guessing last week, so we score him one

Consultant Yeah erm I mean, I think that this is indicating getting to know him rather than any deterioration

Here it is clear that the scores themselves fundamentally depended on team member's knowledge of the patient, which itself changed over time. Initially, team members underestimated the severity of the patient's problems, the true extent of which only came to light as they got to know him better. In reality, the severity of Mr. Smith's problems did not change during the first two weeks of his stay in the unit, but his scores alone would suggest that he had deteriorated. Thus, outsiders, without any clinical knowledge of the patient, would gain an inaccurate impression of the patient's change over time during rehabilitation, rendering the scores unreliable. However, team members, on the basis of their clinical knowledge of the patient, were able to dismiss the patient's early score as being inaccurate.

### Patient variation and complexity

'Problematic scoring' also occurred because the complexities of patients' problems meant that it was often difficult to identify the root cause of their difficulties and thus to score it. As the speech therapist explained:

"Yes because I think sometimes it's put there about confusion and concentration and sometimes if people have dysphasia it's quite hard to tell whether they've got memory problems and confusion and things because it's just hard to assess when somebody can't find the right words you don't know whether it's a word finding problem or that they're confused." (Speech therapist, interview)

This translated into uncertainty when scoring these problems within MDT meetings, particularly when the patient was newly admitted. This was evident on the second occasion the team scored Mr Taylor, a 60 year old man who had suffered a stroke

SHO Confusion?

OT I'd say two but it's hard to say again because it might be because language is... it's hard to say.

SHO Erm concentration?

OT Four.

SHO Memory?

OT I reckon four but again it's hard to say

A further source of difficulty was that the patients themselves varied in their ability to perform tasks from one day to the next, making it difficult to get a consistent picture of what they were able to do. Despite this, the nurse maintained that, overall, the scores were an accurate reflection of the patient. The nurse explained

"I think they are fairly accurate. Not, as I say, it's different because the patients do vary a lot, you know, between from week from week, they do differ. You know, one day they could be having an off day like we all do. We all have off days and we don't quite do as much as we do say other days"

Team members also reported that patients' ability to perform particular tasks also varied across settings. The Barthel Index specifies that patient should be scored on what they are *able *to do, rather than what they are *potentially *able to do. In applying the Barthel Index in practice, team members specified that the score should be based on what patients were able to do on the ward, rather than in therapy. However, in these instances the nurse explained that disagreements about the score were more likely to happen:

"if the patient's different on the ward to say in physio or OT. They could do a perfect transfer in say physio, but then on the ward they might not be very good, you know. I don't know whether it's because they perform better for the physios. Sometimes you know it does happen. It does vary".

Again, this variability led to uncertainty in allocating a score to the patient within team meetings, as can be seen below when the team were scoring Mrs Slater, two months after her admission to the unit following a stroke.

Registrar Dressing?

OT I'm going to keep it the same score but I would query that it's actually that high to be honest because she does need a lot...

Registrar Is it eight?

OT Ermmm....

Consultant What do you think it really is?

OT Well I'd....

Registrar even if you fill....?

OT I'd say it was five actually but I want to assess further because whether you know may, maybe she had a bad day and a....

Registrar mmm mmm ok (quietly) Personal hygiene (louder).

OT and a three for personal hygiene rather than a 4 or 5

Registrar three?

OT but again I'll have another

Thus, difficulties in identifying the cause of the patient's problems and patient variability over time meant that scores were provisional and open to further revision, rather than fixed and certain. The reliability of the scores was not a 'fixed' property of the measure itself, but was dependent on a range of contextual factors when applied in practice.

### Complexity of the concepts to be scored

'Unproblematic scoring' was more likely to occur for the Barthel Index and, in particular, the items scored by the physiotherapist (ambulation, transfers, wheelchair use and ability to climb stairs). These scores were subject to little overt disagreement or questioning and any uncertainty was usually resolved by the physiotherapists themselves. This was attributed to the clarity of the scoring guidelines for these items, as one of the physiotherapists explained in her interview:

"I mean for our, for our bit the parameters are quite clearly defined scores so it's fairly easy to score, the parameters are set really certainly for ours, you know you can and it tends to be what we do on the ward, it might not be what you want them to do or what they're capable of doing but what they actually do...But yes for us certainly the parameters are like where you, what score you give them are quite clear really" (Physiotherapist)

'Problematic' scoring was more likely to occur for the 'homegrown' single item measures of memory, concentration, confusion, drive/motivation, depression, anxiety and behaviour, and for the handicap items measuring orientation and social integration. For the 'homegrown' items, these difficulties may have been due to the more limited information provided in the scoring guidelines for these items. Certainly, one consultant described them as "a very crude categorisation" while the other consultant considered them to be "very basic, not sophisticated" and acknowledged that they were "somewhat inconsistent in the way we score that" referring in particular to the depression and anxiety scores. However, there were detailed scoring guidelines for the items within the handicap measure, which had been subjected to formal psychometric assessment and yet uncertainty and discussion about the scores on these items were still evident. A number of further issues may also account for this.

The concepts addressed by the 'homegrown' scores and the two items in the handicap measure are inherently more difficult to define and therefore measure than the patient's ability to transfer from a bed to a chair, or their ability to walk upstairs (examples of items from the Barthel Index). Thus, practitioners from different professional backgrounds may define and understand these concepts differently. Colombo et al [34] identified that practitioners from different professional background differed in the ways they understood and defined mental health problems. Deficits in the patient's memory, concentration or orientation; behavioural problems and symptoms of depression or anxiety are likely to become apparent during the course of every team member's work with the patient. However, due to the different ways in which team member's worked with patients, they may become manifest in different ways. As the nurse explained "with depression, for instance, then the psychologist on the ward, she might see a different side of the anxiety and she may have another impression" compared to the nursing staff.

Taken together, these characteristics of the 'homegrown' scores and items on the handicap measure suggests that coming to a shared understanding about the meaning of these scores and how to rate their severity was more likely to require negotiation and discussion amongst team members. This is illustrated in the extract below, where the team is trying to agree on a 'confusion' score for Mr Fanshawe, a patient who had been admitted to the unit three weeks previously with a subarachnoid haemorrhage

SHO1 Confusion?

OT Because he hasn't changed score so I guess (inaudible) which is I guess four, four, memory two, drive three....

Consultant I think I, he's better than that.

Nurse Mmm, yeah.

Physio Mmmm, I would

Consultant I don't think that seems right anymore.

(Pause)

OT Uuuumm (looking through her notes)

Nurse His memory

OT He's significant cognitive memory mood and (inaudible) behaviour

(Inaudible)

Consultant He was able to give us a very clear account of his weekend at home.

OT Right.

Nurse I think because he's back, back to familiar surroundings again has actually helped him that, that much better really.

OT Ok, well I, I mean I don't know the guy so. I think otherwise...

Consultant What's our consensus here? The memory score is down the bottom there isn't it?

SHO Yeah, erm

Nurse The man can do his national insurance number spot on so that's...

Consultant That's pretty good.

OT I don't even remember mine! (Laughs)

SHO Erm, so at the moment he's got moderate erm memory impairment ... but we could say it is slight affecting progress and slight not affecting progress.

Consultant I don't think its affecting progress is it?

Nurse I think when I was saying on Tuesday though that he refused to acknowledge that he did have a drink problem considering that he lay in bed.

FLHV = When I saw him last week and spoke to him he was hiding a lot of things, either that or just doesn't want to remember ...and the social worker said there is a lot more going on at home. She is going to come into the family meeting this month to highlight, to give us (inaudible).

Consultant I'm not sure that's memory problem though.

Nurse No.

SHO No.

OT Insight

FLHV Insight would be it wouldn't it, in commas.

Consultant Yeah I think it, I think he knows (slight laugh) but he's not saying.

SHO Shall I give him a four then, slight not affecting?

Consultant Yeah, yeah

Debate surrounds the meaning of the terms 'moderate memory problems' (a score of two) and 'slight memory problems' and whether the patient's 'slight' memory problems can be construed as 'affecting progress' in rehabilitation (a score of three) or 'not affecting progress' (a score of four). Team members attempted to make sense of these terms by offering examples of the patient's behaviour which may be indicative of a memory problem and a reflection of its severity. Although a consensus on the score was reached in the end, there was sometimes disagreement between team members as to what constitutes a memory problem, as can be seen when the consultant did not agree that the examples offered by the nurse and the family liaison health visitor could be defined as a memory problem.

Such debates occurred even when there were scoring guidelines available to assist with interpretation. In the extract below, the team are scoring the 'social integration' item on the handicap measure for a new patient, Mr Edwards, a 40 year old man who had suffered a severe head injury resulting in cognitive problems.

Registrar And social integration, seven he doesn't really (inaudible)

OT Socialise

Registrar He doesn't really interact with anyone else does he?

SALT He does, he

Physio He does, he does, yes.

SALT (inaudible)

Physio Yeah he'll be a six won't he, do you think he'll be a six, disturbed. (pause) [reading scoring guidelines]. Can't say behavioural problems prevent co-existence and cannot develop social, what do you think he's

SALT I don't know, essentially he'll, you know

Physio Chat away

SALThe can have a chat

Physio A chat, but you can't say he fully relates

Nurse = He can't quite do a conversation

Physio so he must be difficulty in relating effectively with significant others, also (inaudible), he's not a five though is he,

SALT = No, no, no

Physio he doesn't fully relate socially to like (inaudible) does he

Registrar So six we're saying

The team are trying to decide if the patient is 'unable to relate to others [as] behavioural problems prevent co-existence [or] cannot develop social relationships due to impairment or disability' (a score of seven), or has 'difficulty relating effectively to significant others' (a score of six) or 'only fully relates socially to one other eg spouse' (a score of five). It is possible to see two sources of uncertainty here. The first rests on whether the patient does or does not interact with others. The physiotherapist and speech therapist argued that he does but the specialist registrar believed that he does not. It is likely that these differing perspectives relate to the different degree of contact that team members have had with the patient and in different contexts. Secondly, it was also unclear to team members whether having a 'chat' constitutes 'relating' to others. Even where scoring guidelines were present to assist with interpretation, discussion and negotiation were required to agree on a shared meaning of the terms. As others have also found, scoring guidelines were not always sufficient to address scoring problems [33]; scoring guidelines themselves always require interpretation.

## Discussion

This paper aimed to examine how multidisciplinary teams achieve the task of scoring patients on a series of standardised outcome measures and explore the challenges they faced. Given the case-study approach used, any claims to the generalisability of our findings must be limited. Our data collection was confined to the process of scoring standardised measures within MDT meetings and we recognise that other forms of scoring measures, particularly those used by individual clinicians, were missed. Despite this, the observational techniques used were invaluable in providing a rich descriptive and narrative account of the ways in which scoring patients on standardised outcome measures by multidisciplinary teams was achieved in practice. The site we observed was purposively sampled, and (so far as the authors are aware) was broadly typical of similar units elsewhere in the English NHS. Further research is needed to determine whether our findings apply to other settings and to other standardised measures.

The production of the scores themselves was determined by the social organisation of work within the multidisciplinary team. As the knowledge to produce the scores was distributed amongst different professions within the team, different team members were responsible for producing a score for different sets of items, depending on their professional background. This is in contrast to how scores are produced in a research context, where they are usually the responsibility of a single, independent observer. In many instances, this division of labour was sufficient to bring together team members' knowledge to score the patient in a consensual manner, giving rise to 'unproblematic' scoring. In other cases it was not sufficient and scoring the patient became problematic. In problematic scoring, the scores were uncertain and subject to revision and adjustment. They sometimes required negotiation to agree on a shared understanding of concepts to be measured and the guidelines for scoring. From a psychometric perspective these problems would raise questions about the validity, reliability and responsiveness of the scores. However, from a clinical perspective, such characteristics are an inherent part of clinical judgement and reasoning [47,48].

We identified a number of issues that gave rise to this problematic scoring. Team members' knowledge of the extent of patient's problems changed over time, which meant that initial scores sometimes did not reflect the true extent of these problems and were revised or dismissed, creating an impression of deterioration when none had occurred. Patients had complex problems which could not easily be distinguished from each other and patients themselves varied in their ability to perform tasks from one day to the next creating uncertainty about the reliability of the scores. These difficulties were further compounded by team members from different professions working in patients in different ways, which led to team members having different but equally valid perspective on patients' problems. This was a particular issue when scoring concepts such as anxiety, depression, orientation, social integration and cognition. These concepts were inherently difficult to define but disagreements may reflect differences in working models of health and illness the different team members held by virtue of their professional training and day to day work with patients. Thus, Anspach's [24] conceptualisation of the organisation as an 'ecology of knowledge' is a useful way of understanding some of the challenges that occurred in arriving at a shared understanding of the concepts to be scored.

Concern has been expressed that clinicians have a vested interest in the outcome of an intervention and so may not be able to rate outcome measures objectively [20]. We did not find any evidence of self serving biases in the ways that team members scored the measures; for example, the speech therapist did not appear to be inclined to exaggerate the patient's communication abilities, nor did the physiotherapists overstate the quality of the patient's movement. Instead, as we have noted elsewhere [46] there was an implicitly accepted 'rule' to score the patient at the lowest score if there was any doubt or uncertainty about their abilities, especially at the beginning of the patient's rehabilitation. The mobilisation of this rule, however, was often context dependent and either served to maximise the patient's improvement during rehabilitation for the purposes of providing aggregated data to the local PCT, or served to minimise the appearance of change when the scores were used to discuss a lack of progress in the patient with family members [46]. Such 'moulding' of the scores was deemed legitimate by team members to ensure that the scores accurately reflected clinical opinion, but 'outsiders' could construe this as bias.

Thus, our findings suggest that the application of standardised outcome measures in clinical practice may depart in important ways from the ways in which they are applied in the relatively controlled studies in which the psychometric properties of the measure are established and in which the measure is then used to evaluate the effectiveness of treatments in research studies. Establishing the psychometric properties of an instrument is acknowledged to be an ongoing project, but these properties tend to be seen as 'fixed' for a particular population and setting once the measure is deemed to meet a set of criteria. We would argue that, in clinical practice, it is more helpful to view the psychometric properties of an instrument, like other aspects of clinical judgement, as a local accomplishment amongst social actors [47], rather than as an 'inherent' or 'fixed' property of the measure for a particular population and/or setting. Although research studies may provide evidence to support the reliability, validity and responsiveness of an instrument, such properties cannot be seen as residing within the measure itself, but are socially accomplished and context dependent.

## Conclusion

It has been argued that outcome measures designed in research settings are not sufficiently precise to be used for the reliable monitoring of individual patients [7]. This study would suggest that when standardised outcome measures are applied in real life clinical practice, their reliability, validity and responsiveness to change may not match that found in research studies assessing these psychometric properties or when they are applied in research settings. However, this has to be set in the context of how clinicians actually use and interpret such measures in clinical practice, as opposed to their use in research contexts. We found that uncertainty about score allocation prompted a need for discussion amongst team members to agree on a shared understanding of the concepts to be measured. 'Scoring' patients was the means through which information about patients' problems could be shared, discussed and negotiated amongst team members. Thus, revisions and adjustments to the scores and negotiations about meaning are integral to the process of using and interpreting standardised outcome measures in clinical practice, rather than merely sources of error. Furthermore, as we have shown elsewhere [46], clinicians do not apply such measures in their decision making in a 'cookbook' fashion. They employ tacit knowledge to interpret the significance of changes in the scores over time and to balance the importance of information from the scores against other clinical and social knowledge about the patient. The scores rarely form the basis of decisions about patients but instead act as a support to those decisions. Thus, while we think it is important to highlight the challenges faced by multidisciplinary teams in scoring patients on standardised outcome measures, it would be unwarranted to conclude that such challenges imply that these measures should not be used in clinical practice to facilitate decision making about individual patients.

Nevertheless, our findings do raise some concerns when the routine collection of clinician rated standardised outcome measures is used to support performance management, benchmarking and assessments of service or treatment effectiveness [15,16]. Interpreting such data is also made problematic by the need for adjustment of confounding factors that may modify the relationships between process and outcome, which may further compound inaccuracies created through their collection in routine clinical practice [49-51]. While clinical judgement and tacit knowledge can assist with the interpretation of standardised outcome measurements for individual patients, this knowledge is not applicable to population data since the link to individual patient circumstances and the context in which the data were collected is lost. This reinforces the importance of making decisions about the uses to which standardised outcome measures are to be put before embarking on their collection. The findings of the study question whether the routine collection of standardised outcome measures can play a role in the decision making about individual patients *at the same time *as providing data for the purposes of performance management. Future research is needed to explore these tensions and examine how outcome data, collected in clinical practice is or can be used in such performance management activities.

## Competing interests

The authors declare that they have no competing interests.

## Authors' contributions

JG, ST, AFL and RF designed the study; JG collected the data; JG, RF, AFL and ST analysed the data, JG drafted the manuscript, RF, AFL and ST critically reviewed and provided comments on the manuscript.

## Pre-publication history

The pre-publication history for this paper can be accessed here:


